# Agricultural waste-derived activated carbon/graphene composites for high performance lithium-ion capacitors

**DOI:** 10.1039/c9ra04721b

**Published:** 2019-09-17

**Authors:** Bing Li, Hongyou Zhang, Cunman Zhang

**Affiliations:** Clean Energy Automotive Engineering Center, Tongji University Shanghai 201804 China libing210@tongji.edu.cn +86 21 69589355 +86 21 69589355; School of Automotive Studies, Tongji University Shanghai 201804 China

## Abstract

Agricultural waste, corncob-derived activated carbon (AC) is prepared by pre-carbonization of the precursors and activation of KOH of the pyrolysis products. The AC oxidized by HNO_3_ is called OAC. The OAC/reduced graphene oxide (rGO) composites are prepared by urea reduction (aqueous mixture of OAC and graphene oxide). The influence of the mass ratio of graphene oxide (GO) on the electrochemical properties of OAC/rGO composites as electrode materials for electrochemical capacitors is studied. It is found that the rGO sheets were used as a wrinkled carrier to support the OAC particles. The pore size distribution and surface area are dependent on the GO mass ratio. In addition, the rate capability of OAC is improved by introducing GO. For the OAC/rGO composites prepared from precursors with a GO mass ratio of 5%, the best rate performance was achieved. The lithium ion capacitor, based on OAC/rGO as cathode and Si/C as anode, exhibits a high energy density of 141 W h kg^−1^ at 1391 W kg^−1^. 78.98% capacity retention is achieved after 1000 cycles at 0.4 A g^−1^.

## Abstract 2

Agricultural waste, corncob-derived activated carbon (AC) is prepared by pre-carbonization of the precursors and activation of KOH of the pyrolysis products.

## Graphical abstract

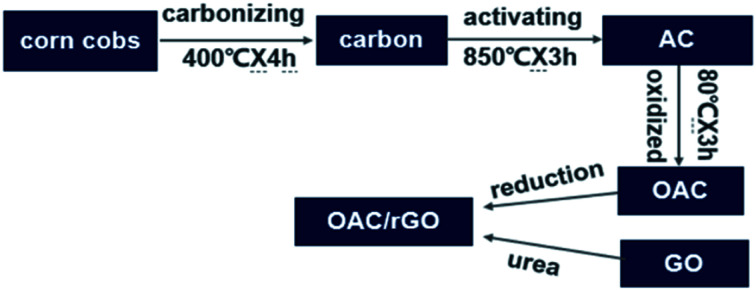

## 1. Introduction

In recent years, with the increasingly prominent energy and environmental issues, the rapid development of electric vehicle smart grids and energy storage technologies, people have put forward higher requirements for energy conversion equipment. Due to their excellent performance, supercapacitors and lithium-ion batteries are currently recognized as two promising systems. Lithium-ion batteries (LIBs) have the advantages of high energy density, high operating voltage, no memory effect, but low power density and short cycle life. Supercapacitors (SCs) have higher power density and longer cycle life, but their energy density is low, only 1/10 of lithium-ion batteries. Lithium-ion capacitors (LICs) are composed of a capacitor-type electrode and a battery-type electrode. LICs exhibit higher power performance, longer life cycle, and a wider temperature window than LIBs. Compared to SCs, the energy density of LICs is several orders of magnitude higher. So LICs provide a bridge between LIBs and SCs for many applications.^1,2^

Because of excellent thermal and chemical stability, large specific area, and relatively low cost, porous carbons are usually used as electrode materials for LICs activated carbon/graphene composites with high-rate performance as electrode materials for electrochemical capacitors.^1,3,4^ Activated carbon,^3,5–7^ templated carbon,^8,9^ carbon nanotubes,^10,11^ and graphene^1,3,12^ have been widely studied as the electrode materials for LICs. Due to its excellent electrochemical performance and easy to achieve large-scale production, AC is still used as an electrode material in the industry. Coal^13–15^ is the most widely used precursor in AC production, but it is a good complement to natural bio-sources and polymer production ACs due to energy requirements. Due to its special composition and structure, easy large-scale production and low price, agricultural waste (peanut shell, banana peel, *Prosopis juliflora*,^16^*etc.*) are particularly attractive precursors for AC. However, conventional AC exhibits a low electrical conductivity.

Chemically modified graphene^17^ is a new type of carbon material with good electrical conductivity and mesoporous structure. It is an ideal filling material for microporous carbon. The research on AC/rGO composites for LIC is very limited. There have been only a few reports on the performance of AC/rGO composite LIC so far.^3,18^ But the method of sol–gel takes a long time and is harmful to the environment. Low cost and environment friendly hydrothermal method are considered to be more competitive method for the preparation of AC/rGO.

In this paper, we report a novel route for the preparation of AC/rGO composites utilizing agricultural waste (corncob) as a precursor and study the impact of the mass ratio of the introduction of rGO into OAC on the electrochemical performance.

## 2. Experimental

### 2.1 Preparation of OAC/rGO

**Fig. 1:**
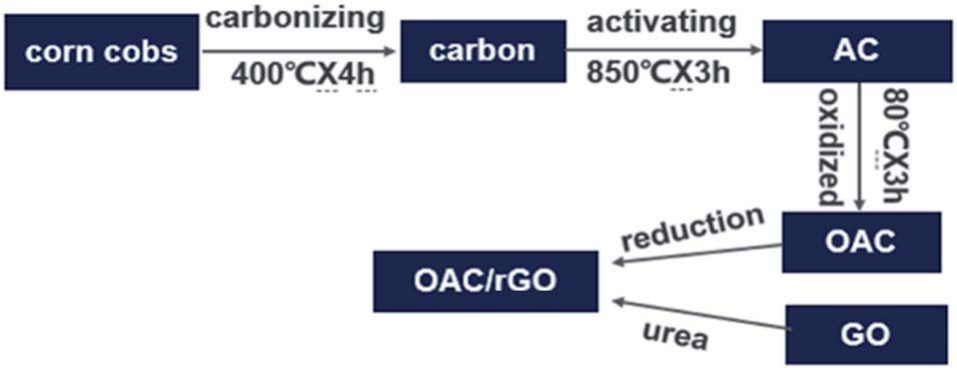
Schematic diagram of preparation of OAC/rGO.

The preparation process of OAC/rGO is shown in Fig. 1. Carbon was first prepared by carbonizing the most common agricultural waste corn cobs. Corncob is derived from Chifeng, Inner Mongolia. Subsequently, the product was chemically activated by KOH as an activator at 850 °C for 3 h. The detailed process is reported in our previous research.^19^ There was an additional ball milling process after activation. The AC is oxidized by HNO_3_ at 80 °C for 3 h. The OAC/rGO composites are prepared by urea reduction (aqueous mixture of OAC and GO). The obtained finer powders were used to fabricate the electrode. A sample of *X*% graphene oxide content was named OAC/rGO *X*%.

### 2.2 Characterization

The morphologies and texture analysis of the samples were performed by scanning electron microscopy (SEM) and transmission electron microscopy (TEM). The microstructure of the sample was analyzed using an ASAP 2020 spectrophotometer. The elemental contents and surface functional groups were determined by elemental analysis, Raman spectrums and X-ray photoelectron spectroscopy (XPS). A micromeritics ASAP 2020 sorptometer was used to analyze the microstructure of the samples.

### 2.3 Electrochemical measurements

The prepared OAC/rGO was mixed with Super-P carbon black and Na-alginate binder (8 : 1 : 1) in de-ionized water to form a uniform slurry, which was coated on aluminum foil. The electrodes were separated using a glass microfiber filter (Whatman GF/C) impregnated with electrolyte. In the measurement of the half-cell, we used metallic lithium as the reference and counter electrode. In LIC, OAC/rGO is used as the cathode and Si/C is used as anode in 1 M LiPF_6_ dissolved in dimethyl carbonate and fluoroethylene carbonate (DMC : FEC = 4 : 1 by vol).

The LICs were also assembled in coin cells with Si/C anode (pre-cycled for 1 cycle at 0.1C) and OAC/rGO cathode in the same electrolyte, and the optimized mass ratio of cathode and anode was 1 : 1. All the electrochemical tests were carried out at room temperature. The voltage range of Si/C half-cell was 0.01–1.0 V. The OAC/rGO electrodes and the LIC were measured at the same voltage range of 2.0–4.5 V. The energy density and power density were calculated based on the total mass of active materials on both the anode and cathode. Cyclic voltammetry (CV) was tested at a scan rate ranging from of 5 to 20 mV s^−1^ (2.0 to 4.5 V). A constant current charge and discharge cycle was performed at a current density of 0.1–0.4 A g^−1^. Electrochemical impedance spectroscopy (EIS) was tested in a frequency range of 0.1 Hz to 100 kHz with a potential amplitude of 10 mV. All experiments were performed using a CHI760E electrochemical workstation. The cycle performance was measured under normal temperature conditions using a LAND battery tester (Wuhan Jinnuo Electronics Co., Ltd.).

## 3. Results and discussion

**Fig. 2:**
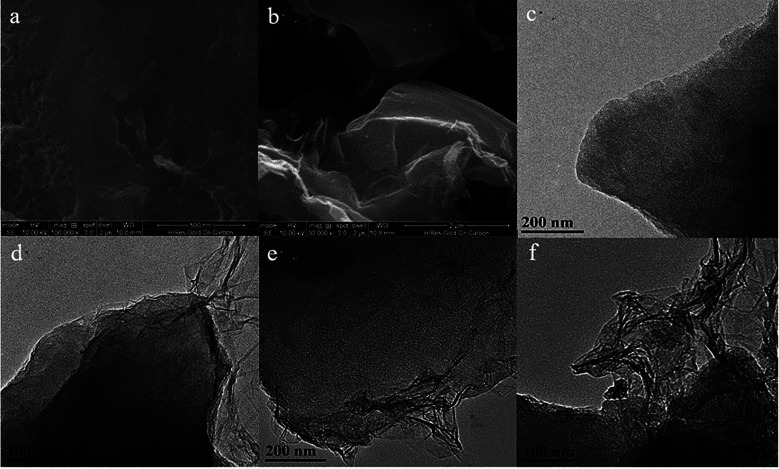
SEM photograph of OAC (a) and OAC/rGO 5% (b), TEM image of OAC (c), OAC/rGO 2% (d), OAC/rGO 5% (e) and OAC/rGO 8% (f).

Fig. 2 shows the SEM photographs of OAC and OAC/rGO 5%, TEM image of OAC, OAC/rGO 2%, OAC/rGO 5% and OAC/rGO 8%. As can be seen from the figure, the AC samples are composed of a plurality of irregular micron-sized crushed particles while partially maintaining the original honeycomb structure of the corn cob. The combination of the original honeycomb channel of the corn cob and the multi-stage pore of the nanopore structure can provide rapid transport of ions while increasing the specific surface area of the material, thereby effectively increasing the specific capacity. After the addition of graphene oxide, the structure did not change much, and the original characteristic structure was maintained. As the graphene oxide content increases, the carbon surface is covered with a thin layer of graphene oxide. However, as the graphene oxide content continues to increase, graphene oxide itself agglomerates and entangles.

**Fig. 3:**
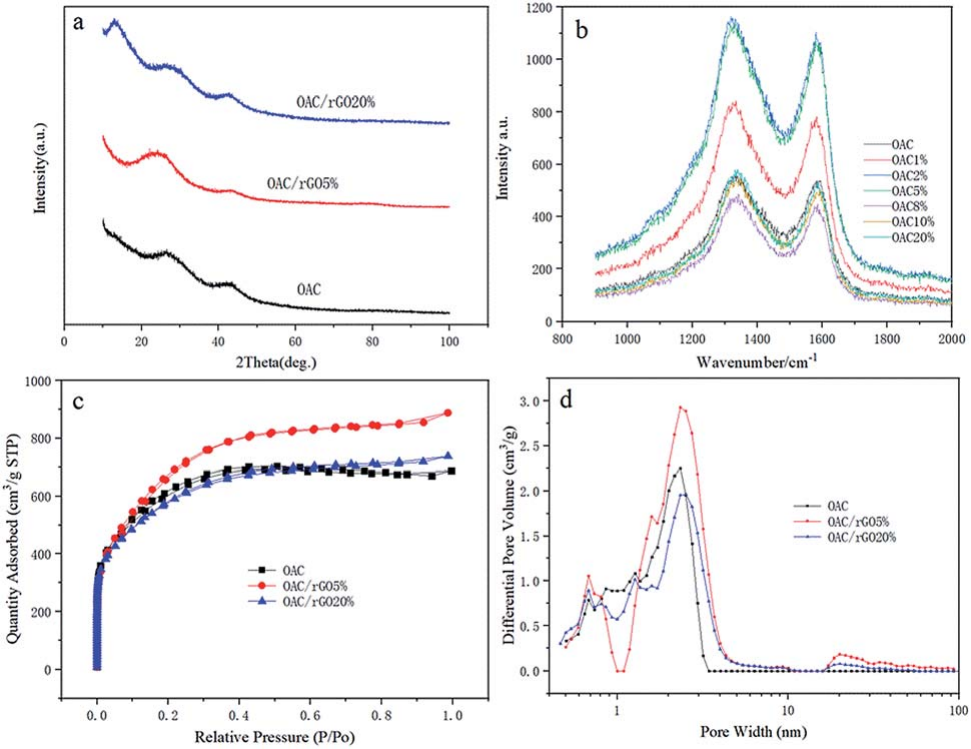
(a) X-ray diffraction patterns of OAC, OAC/rGO 5%, OAC/rGO 20%; (b) the Raman spectra of samples of different contents of graphene oxide; (c) nitrogen adsorption–desorption isotherms and (d) micropore size distribution of OAC, OAC/rGO 5%, OAC/rGO 20% evaluated by HK method.

XRD analysis results for OAC, OAC/rGO 5% and OAC/rGO 20% are shown in Fig. 3a. All samples of the X-ray diffraction pattern were observed between the broad diffraction peaks of 10° and 45°, indicating that the as-prepared sample was in an amorphous state. As the mass ratio of rGO in the precursor increases, the plane diffraction peak shifts to the right, which may be related to the change of surface microstructure caused by the introduction of rGO. The surface structure strongly influences the formation of the electrochemical double layer, which in turn determines the rate capability of the electrode material. XRD analysis results for OAC, OAC/rGO 5% and OAC/rGO 20% are shown in Fig. 3a. The Raman spectra of samples of different contents of graphene oxide is shown in Fig. 3b. In the Raman spectrum, the small peaks at 1340 and 1590 cm^−1^ correspond to the D and G peaks of carbon, respectively. The peak intensity ratio *I*_D_/*I*_G_ of the two peak shapes is about 1, further demonstrating that the AC/rGO composite mainly exists in an amorphous form and is partially graphitized. The nitrogen adsorption–desorption isotherms at 77 K and the pore size distribution of the obtained OAC, OAC/rGO 5%, and OAC/rGO 5% samples are shown in Fig. 3c and d. Obviously, the meso/macro pores is good for mass transfer, thus ensuring the power performance of the capacitor.^20,21^ In addition, the presence of sufficient micropores can effectively increase the active sites of charge storage. The N_2_ adsorption–desorption isotherm obtained a corn-based OAC/rGO 5% BET specific surface area of 2553 m^2^ g^−1^, confirming the presence of micropores. As can be seen from the PSDs chart, the micropore volume is 1.35 cm^3^ g^−1^.

**Fig. 4:**
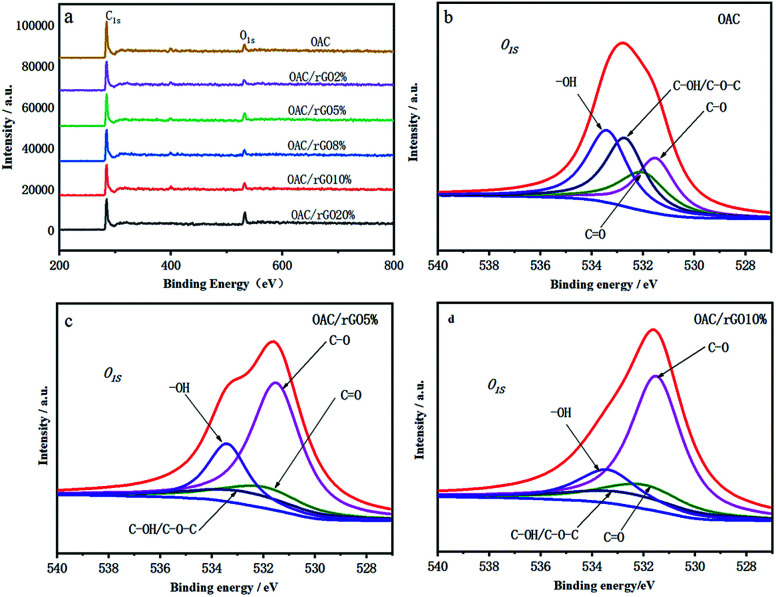
(a) XPS spectra of samples of different contents of graphene oxide; (b)–(d) deconvolution results of O 1s peaks for OAC, OACrGO 5%, and OACrGO 10%.

The C, N and O content of samples was showed by elemental analysis in Table 1. As the content of graphene oxide increases, the oxygen content decreases from 12.17 wt% for OAC to 5.47 wt% for OAC/rGO 8%. Rich surface oxygen distribution was tested by XPS analysis in Fig. 4. The XPS spectra of the O 1s region can be approximately fitted into four main peaks corresponding to oxygen atoms in C=O groups,^21^ oxygen atoms in C– O groups in C–OH and/or COOR,^20^ oxygen in C–OH/C–O–C groups and oxygen in –OH groups.^22,23^ These types of surface oxygen functional groups have the influence on the capacity of the engineered carbon for their fast reaction with lithium.^24^

**Table 1:** Composition of different samples

Sample	Composition		
	C%	N%	O%
OAC	72.21	0.85	12.17
OAC/rGO 2%	70.33	3.88	6.09
OAC/rGO 5%	69.67	3.71	6.56
OAC/rGO 8%	50.28	2.84	5.47

**Fig. 5:**
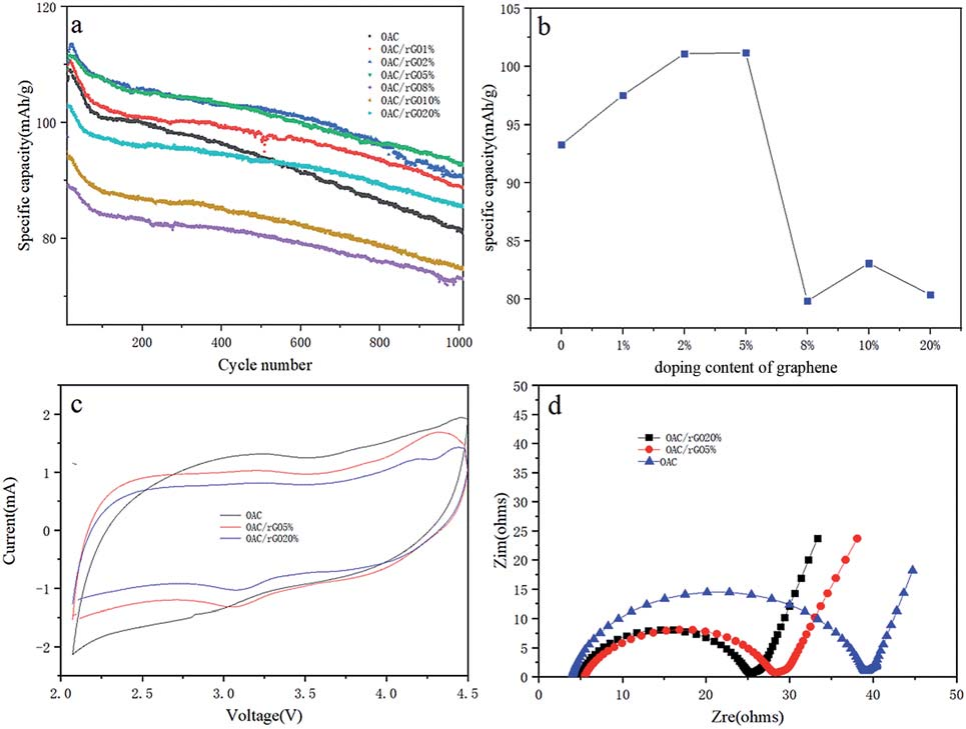
(a) The charge–discharge curve at 0.4 A g; (b) the average capacity change of 1000 cycles of OAC/rGO composites with different contents of graphene oxide; (c) CV diagram of three materials at scan rate of 5 mV s; (d) EIS diagram of three materials.

It is essential to evaluate the single-electrode performance along with metallic lithium to determine the mass loading. The OAC/rGO cathode was tested in a half cell from 2 V to 4.5 V *vs.* Li (current density of 0.4 A g^−1^), and the corresponding results are presented in Fig. 5a. The OAC/rGO 5% shows high initial specific capacities and high cycle capacity retention rate. Fig. 5b shows the average capacity change of 1000 cycles of OAC/rGO composites with different contents of graphene oxide. It can be seen that as the content of graphene oxide increases, the average capacity first increases and then decreases. Which is consistent with the previous physical characterization results. When the graphene oxide content is not high, it improves the conductivity and enhances the stability of the cycle. Continued increase will cause agglomeration to block the pore structure of the activated carbon resulting in a decrease in capacity due to that the specific capacity of OAC is higher than the specific capacity of graphene. Fig. 5c shows the CV curves of electrodes of OAC, OAC/rGO 5% and OAC/rGO 20%. As shown in Fig. 4c, the CV curve of OAC is a typical quasi-rectangular shape, indicating that the electrochemical performance of the OAC electrode is mainly affected by the double layer capacitance. As the proportion of rGO in the composite increases, obvious redox peaks appeared. It indicated that the doping of graphene oxide introduces the oxygen-containing functional group participating in the reaction during charge and discharge. In order to clarify the improvement of the performance of OAC/rGO composites, the electrochemical impedances of OAC, OAC/rGO 5% and OAC/rGO 20% electrodes were measured using a two-electrode configuration. The Nyquist plot of the electrode material is shown in Fig. 5d. The equivalent series resistance (ESR) reflects the resistance to electron conduction and ion transport in the electrochemical system. As shown in Fig. 5d, as the mass ratio of rGO increases, the ESR of the electrode gradually decreases. Therefore, the introduction of rGO not only reduces the resistance of the electrode, but also promotes the migration of ions in the electrode material. As the rGO load in the composite increases, the rate performance of the composite increases.

**Fig. 6:**
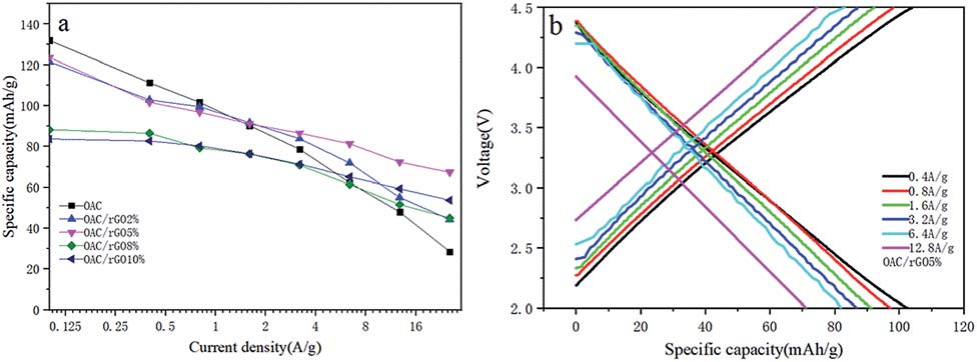
(a) Average capacity change of OAC/rGO composites with different graphene oxide contents at different current densities; (b) voltage profile of OAC/rGO 5% at different current densities.

To further investigate the rate performance of the OAC/rGO electrodes, we measured the rate capability of seven samples at different current densities, as shown in Fig. 6a. The OAC/rGO cathode materials with different graphene oxide contents are tested at the currents of 0.1 A g^−1^, 0.4 A g^−1^, 0.8 A g^−1^, 1.6 A g^−1^, 3.2 A g^−1^, 6.4 A g^−1^, and 12.8 A g^−1^ for 10 cycle. It can be seen from the figure that the cycle stability of OAC/rGO 5% of graphene oxide is the best. For AC/rGO 5%, the capacity of 140 mA h g^−1^ is achieved at a current density of 0.1 A g^−1^. When the current is increased to 12.8 A g^−1^. The AC/rGO 5% provides 75.11 mA h g^−1^. Which is higher than OAC/rGO composites with other contents of graphene oxide. The linear constant current charge/discharge curve of OAC/rGO 5% was tested at 0.4–12.8 A g^−1^, indicating that the adsorption/desorption of ions on the electrode surface is capacitive, as shown in Fig. 6b.

**Fig. 7:**
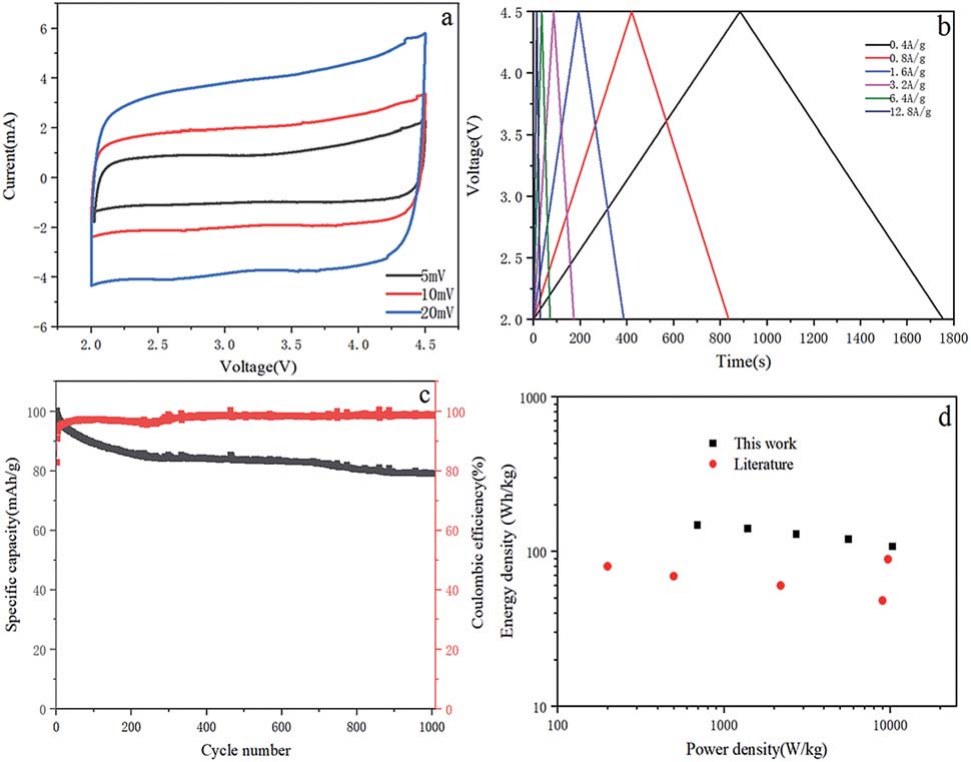
(a) CV diagram of the OAC/rGO 5%//Si/C LIC full cell; (b) voltage profile of the OAC/rGO 5%//Si/C LIC; (c) long cycling performance of the OAC/rGO 5%//Si/C LIC at 0.4 A g; (d) Ragone plot of the OAC/rGO 5%//Si/C LIC.

The full cell is composed of OAC/rGO 5% as the cathode and Si/C as the anode. During the charging process, the PF_6_^−^ ions are absorbed by the porous structure of the OAC/rGO 5%, while the Li^+^ electrolyte ions are alloyed with the Si/C anode. The discharge process is the opposite of the charging process. In order to obtain the best electrochemical performance and energy/power density, the mass ratio of the electrode active material was optimized to 1 : 1. Due to the superposition of two different energy storage mechanisms, the CV curve of the LIC gradually deviates from the ideal rectangular shape as the scanning speed increases (Fig. 7a). This observation is consistent with the voltage carves of LIC, which has little deviation from the linear slope (Fig. 7b). The LIC maintains a capacity of 78.98% in 1000 cycles at a current density of 0.4 A g^−1^ and has good cycle stability (Fig. 7c). The coulombic efficiency of the full cell is relatively high with an average of 98.1%. The Ragone plot of LIC (power density and energy density, material grade) is shown in Fig. 7d. The energy density and power density are calculated from the total mass of active material on the cathode and anode. The LIC has an energy density of 141 W h kg^−1^ at the power of 1391 W kg^−1^, and its energy density remains at 108 W h kg^−1^ even when the power density is increased to 10 299 W kg^−1^. Compared with other LIC systems with typical energy and power density, such as AC//hard carbon,^25^ AC//soft carbon,^26^ AC//LTO^16,27^ and AC//B–Si/SiO_2_/C.^28^ The performance of this work is still quite promising for LIC.

## 4. Conclusions

OAC/rGO composite electrode materials for electrochemical capacitors were prepared by pre-carbonization, KOH activation, HNO_3_ oxidation and urea reduction using agricultural waste, corncob and graphene oxide as raw materials. As the mass ratio of the GO increases, the specific capacitance increases first and then decreases. The OAC/rGO 5% composite has the highest specific capacitance. There were no significant differences in the types and contents of oxygen-containing functional groups on the surface of OAC, rGO and their composites. However, the introduction of GO increases the specific surface area and micropore volume of the composite due to the dispersion effect, resulting in an increase in specific capacitance. In addition, the mesoporous volume of the composite material is slightly increased and the rate performance is improved. The LIC was further assembled and evaluated using OAC/rGO and Si/C nanocomposites. After 1000 cycles, LIC has good long-period stability with a capacity retention of 78.98%.

## Conflicts of interest

There are no conflicts to declare.

## Supplementary Material
